# Analysis of nonlinear noisy integrate & fire neuron models: blow-up and steady states

**DOI:** 10.1186/2190-8567-1-7

**Published:** 2011-07-18

**Authors:** María J Cáceres, José A Carrillo, Benoît Perthame

**Affiliations:** 1 Departamento de Matemática Aplicada, Universidad de Granada, E-18071, Granada, Spain; 2 ICREA and Departament de Matemàtiques, Universitat Autònoma de Barcelona, E-08193, Bellaterra, Spain; 3 Laboratoire Jacques-Louis Lions, UPMC, CNRS UMR 7598 and INRIA-Bang, F-75005, Paris, France; 4 Institut Universitaire de France, 75005, Paris, France

**Keywords:** Leaky integrate and fire models, noise, blow-up, relaxation to steady state, neural networks

## Abstract

Nonlinear Noisy Leaky Integrate and Fire (NNLIF) models for neurons networks can be written as Fokker-Planck-Kolmogorov equations on the probability density of neurons, the main parameters in the model being the connectivity of the network and the noise. We analyse several aspects of the NNLIF model: the number of steady states, *a priori* estimates, blow-up issues and convergence toward equilibrium in the linear case. In particular, for excitatory networks, blow-up always occurs for initial data concentrated close to the firing potential. These results show how critical is the balance between noise and excitatory/inhibitory interactions to the connectivity parameter.

**AMS Subject Classification:** 35K60, 82C31, 92B20.

## 1 Introduction

 The classical description of the dynamics of a large set of neurons is based on deterministic/stochastic differential systems for the excitatory-inhibitory neuron network [1,2]. One of the most classical models is the so-called noisy leaky integrate and fire (NLIF) model. Here, the dynamical behavior of the ensemble of neurons is encoded in a stochastic differential equation for the evolution in time of membrane potential v(t) of a typical neuron representative of the network. The neurons relax towards their resting potential $V_{L}$ in the absence of any interaction. All the interactions of the neuron with the network are modelled by an incoming synaptic current I(t). More precisely, the evolution of the membrane potential follows, see [3-8]

(1.1) $$C_{m}\frac{dV}{dt}=-g_{L}(V-V_{L})+I(t),$$

where $C_{m}$ is the capacitance of the membrane and $g_{L}$ is the leak conductance, normally taken to be constants with $\tau_{m}=g_{L}/C_{m}\approx 2\text{ ms}$ being the typical relaxation time of the potential towards the leak reversal (resting) potential $V_{L}\approx -70\text{ mV}$. Here, the synaptic current takes the form of a stochastic process given by:

(1.2) $$I(t)=J_{E}\sum_{i=1}^{C_{E}}\sum_{j}\delta (t-t_{Ej}^{i})-J_{I}\sum_{i=1}^{C_{I}}\sum_{j}\delta (t-t_{Ij}^{i}),$$

where *δ* is the Dirac Delta at 0. Here, $J_{E}$ and $J_{I}$ are the strength of the synapses, $C_{E}$ and $C_{I}$ are the total number of presynaptic neurons and $t_{Ej}^{i}$ and $t_{Ij}^{i}$ are the times of the $j^{\mathrm{th}}$-spike coming from the $i^{\mathrm{th}}$-presynaptic neuron for excitatory and inhibitory neurons respectively. The stochastic character is embedded in the distribution of the spike times of neurons. Actually, each neuron is assumed to spike according to a stationary Poisson process with constant probability of emitting a spike per unit time *ν*. Moreover, all these processes are assumed to be independent between neurons. With these assumptions the average value of the current and its variance are given by $\mu_{C}=b\nu$ with $b=C_{E}J_{E}-C_{I}J_{I}$ and $\sigma_{C}^{2}=(C_{E}J_{E}^{2}+C_{I}J_{I}^{2})\nu$. We will say that the network is average-excitatory (average-inhibitory resp.) if b>0 (b<0 resp.).

 Being the discrete Poisson processes still very difficult to analyze, many authors in the literature [3-5,7-9] have adopted the diffusion approximation where the synaptic current is approximated by a continuous in time stochastic process of Ornstein-Uhlenbeck type with the same mean and variance as the Poissonian spike-train process. More precisely, we approximate I(t) in (1.2) as

$$I(t)\;dt\approx \mu_{c}\;dt+\sigma_{C}\;dB_{t},$$

where $B_{t}$ is the standard Brownian motion, that is, $B_{t}$ are independent Gaussian processes of zero mean and unit standard deviation. We refer to the work [5] for a nice review and discussion of the diffusion approximation which becomes exact in the infinitely large network limit, if the synaptic efficacies $J_{E}$ and $J_{I}$ are scaled appropriately with the network sizes $C_{E}$ and $C_{I}$.

 Finally, another important ingredient in the modelling comes from the fact that neurons only fire when their voltage reaches certain threshold value called the threshold or firing voltage $V_{F}\approx -50\text{ mV}$. Once this voltage is attained, they discharge themselves, sending a spike signal over the network. We assume that they instantaneously relax toward a reset value of the voltage $V_{R}\approx -60\text{ mV}$. This is fundamental for the interactions with the network that may help increase their membrane potential up to the maximum level (excitatory synapses), or decrease it for inhibitory synapses. Choosing our voltage and time units in such a way that $C_{m}=g_{L}=1$, we can summarize our approximation to the stochastic differential equation model (1.1) as the evolution given by

(1.3) $$dV=(-V+V_{L}+\mu_{c})\;dt+\sigma_{C}\;dB_{t}$$

for $V\leq V_{F}$ with the jump process: $V(t_{o}^{+})=V_{R}$ whenever at $t_{0}$ the voltage achieves the threshold value $V(t_{o}^{-})=V_{F}$; with $V_{L}<V_{R}<V_{F}$. Finally, we have to specify the probability of firing per unit time of the Poissonian spike train *ν*. This is the so-called firing rate and it should be self-consistently computed from a fully coupled network together with some external stimuli. Therefore, the firing rate is computed as $\nu =\nu_{\mathrm{ext}}+N(t)$, see [5] for instance, where N(t) is the mean firing rate of the network. The value of N(t) is then computed as the flux of neurons across the threshold or firing voltage $V_{F}$. We finally refer to [10] for a nice brief introduction to this subject.

 Coming back to the diffusion approximation in (1.3), we can write a partial differential equation for the evolution of the probability density p(v,t)≥0 of finding neurons at a voltage $v\in (-\infty ,V_{F}]$ at a time t≥0. A heuristic argument using Ito’s rule [3-5,7-9,11] gives the backward Kolmogorov or Fokker-Planck equation with sources

(1.4) $$\begin{array}{rl} & \frac{\partial p}{\partial t}(v,t)+\frac{\partial }{\partial v}[h(v,N(t))p(v,t)]-a(N(t))\frac{\partial^{2}p}{\partial v^{2}}(v,t)\\ & \;=\delta (v-V_{R})N(t),\;v\leq V_{F}, \end{array}$$

with $h(v,N(t))=-v+V_{L}+\mu_{c}$ and $a(N)=\sigma_{C}^{2}/2$. We have the presence of a source term in the right-hand side due to all neurons that at time t≥0 fired, sent the signal on the network and then, their voltage was immediately reset to voltage $V_{R}$. Moreover, no neuron should have the firing voltage due to the instantaneous discharge of the neurons to reset value $V_{R}$, then we complement (1.4) with Dirichlet and initial boundary conditions

(1.5) $$p(V_{F},t)=0,\;p(-\infty ,t)=0,\;p(v,0)=p^{0}(v)\geq 0.$$

Equation (1.4) should be the evolution of a probability density, therefore

$$\int_{-\infty }^{V_{F}}p(v,t)\;dv=\int_{-\infty }^{V_{F}}p^{0}(v)\;dv=1$$

for all t≥0. Formally, this conservation should come from integrating (1.4) and using the boundary conditions (1.5). It is straightforward to check that this conservation for smooth solutions is equivalent to characterize the mean firing rate for the network N(t) as the flux of neurons at the firing rate voltage. More precisely, the mean firing rate N(t) is implicitly given by

(1.6) $$N(t):=-a(N(t))\frac{\partial p}{\partial v}(V_{F},t)\geq 0.$$

Here, the right-hand side is nonnegative since p≥0 over the interval $\left[-\infty ,V_{F}\right]$ and thus, $\frac{\partial p}{\partial v}(V_{F},t)\leq 0$. In particular this imposes a limitation on the growth of the function N↦a(N) such that (1.6) has a unique solution *N*. Let us mention that a rigorous passage from the stochastic differential equation with jump processes (1.3) to the nonlinear equation (1.4)-(1.6) is a very interesting issue but outside the scope of this paper, see related results in which nonlinearities are nonlocal functionals in [12,13].

The above Fokker-Planck equation has been widely used in neurosciences. Often the authors prefer to write it in an equivalent but less singular form. To avoid the Dirac delta in the right hand side, one can also set the same equation on $\left(-\infty ,V_{R})\cup (V_{R},V_{F}\right]$ and introduce the jump condition

$$p(V_{R}^{-},t)=p(V_{R}^{+},t),\;\frac{\partial }{\partial v}p(V_{R}^{-},t)-\frac{\partial }{\partial v}p(V_{R}^{+},t)=N(t).$$

This is completely transparent in our analysis which relates on a weak form that applies to both settings.

 Finally, let us choose a new voltage variable by translating it with the factor $V_{L}+b\nu_{\mathrm{ext}}$ while, for the sake of clarity, keeping the notation for the rest of values of the potentials involved $V_{R}<V_{F}$. In these new variables, the drift and diffusion coefficients are of the form

(1.7) $$h(v,N)=-v+bN,\;a(N)=a_{0}+a_{1}N,$$

where b>0 for excitatory-average networks and b<0 for inhibitory-average networks, $a_{0}>0$ and $a_{1}\geq 0$. Some results in this work can be obtained for some more general drift and diffusion coefficients. The precise assumptions will be specified on each result. Periodic solutions have been numerically reported and analysed in the case of the Fokker-Planck equation for uncoupled neurons in [14,15]. Also, they study the stationary solutions for fully coupled networks obtaining and solving numerically the implicit relation that the firing rate *N* has to satisfy, see Section 3 for more details.

 There are several other routes towards modeling of spiking neurons that are related to ours and that have been used in neurosciences, see [16]. Among them are the deterministic I&F models with adaptation which are known for fitting well experimental data [17]. General models of this type were unified and studied in terms of neuronal behaviors in [18]. In this case it is known that in the quadratic (or merely superlinear) case, the model can lead to blow-up [19] in the absence of a fixed threshold. We point out that the nature of this blow-up is completely different from the one discussed in this paper. One can also introduce gating variables in neuron networks and this leads to a kinetic equation, see [20] and the references therein. Another method consists in coding the information in the distribution of time elapsed between discharges [21,22], this leads to nonlinear models that exhibit naturally periodic activity and blow-up cannot happen. Nonlinear IF models are able to produce different patterns of activity and excitability types while linear models do not.

 In this work we will analyse certain properties of the solutions to (1.4)-(1.5) with the nonlinear term due to the coupling of the mean firing rate given by (1.6). Next section is devoted to a finite time blow-up of weak solutions for (1.4)-(1.6). In short, we show that whenever the value of b>0 is, we can find suitable initial data concentrated enough at the firing rate such that the defined weak solutions do not exist for all times. We remark that, in the same sense that Brunel in [4], we use the term *asynchronous* for network states for which the firing rate tends asymptotically to constant in time, while we denote by *synchronous* those for which this does not happen. Therefore, a possible interpretation of the blow-up is that synchronization occurs in the model since the firing rate diverges for a fixed time creating possibly a strong partial synchronization, that is, a part of the network firing at the same time. Although one could also consider the blow-up as an artifact of these solutions, since neurons firing arbitrarily fast is not biologically plausible. As long as the solution exists in the sense specified in Section 2, we can get *a priori* estimates on the $L_{\mathrm{loc}}^{1}$-norm of the firing rate. Section 3 deals with the stationary states of (1.4)-(1.6). We can show that there are unique stationary states for b≤0 and *a* constant but for b>0 different cases may happen: one, two or no stationary states depending on how large *b* is. In Section 4, we discuss the linear problem b=0 with *a* constant for which the general relative entropy principle applies implying the exponential convergence towards equilibrium. Finally by means of numerical simulations, in Section 5 we illustrate the results of previous sections about blow-up and steady states. Moreover, this numerical analysis allows us to conjecture about nonlinear stability properties of the stationary states: in case of only one steady state it is asymptotically stable and in case of two different stationary solutions the results show that the one with lower firing rate is locally asymptotically stable while the one with higher stationary firing value is either unstable or with a very small region of attraction. Our results and simulations describe situations which can be identified with neuronal phenomena such as synchronization/asynchronization of a network and bistability networks. Bi- and multi-stable networks have been used, for instance in models of visual perception and decision making [23-25]. Our analysis in Sections 23 and 5 imply that this simple model encodes complicated dynamics, in the sense that, only in terms of the connectivity parameter *b*, very different situations can be described with this model: blow-up, no steady state, only one steady state and several stationary states.

## 2 Finite time blow-up and *a priori* estimates for weak solutions

Since we study a nonlinear version of the backward Kolmogorov or Fokker-Planck equation (1.4), we start with the notion of solution:

**Definition 2.1***We say that a pair of nonnegative functions*(p,N)*with*$p\in L^{\infty }(R^{+};L_{+}^{1}(-\infty ,V_{F}))$, $N\in L_{\mathrm{loc},+}^{1}(R^{+})$*is a weak solution of* (1.4)-(1.7) *if for any test function*$\phi (v,t)\in C^{\infty }((-\infty ,V_{F}]\times [0,T])$*such that*$\frac{\partial^{2}\phi }{\partial v^{2}}$, $v\frac{\partial \phi }{\partial v}\in L^{\infty }((-\infty ,V_{F})\times (0,T))$, *we have*

(2.1) $$\begin{array}{rl} & \int_{0}^{T}\int_{-\infty }^{V_{F}}p(v,t)[-\frac{\partial \phi }{\partial t}-\frac{\partial \phi }{\partial v}h(v,N)-a\frac{\partial^{2}\phi }{\partial v^{2}}]\;dv\;dt\\ & \;=\int_{0}^{T}N(t)[\phi (V_{R},t)-\phi (V_{F},t)]\;dt\\ & \;+\int_{-\infty }^{V_{F}}p^{0}(v)\phi (0,v)\;dv-\int_{-\infty }^{V_{F}}p(v,T)\phi (T,v)\;dv. \end{array}$$

Here, the notation $L^{p}(\Omega )$, 1≤p<∞, refers to the space of functions such that $f^{p}$ is integrable in Ω, while $L^{\infty }$ corresponds to the space of bounded functions in Ω. The set of infinitely differentiable functions in Ω is denoted by $C^{\infty }(\Omega )$ used as test functions in the notion of weak solution. These non-negativity assumptions are reasonable. Indeed, for a given N(t), if we were to replace *N* by $N^{+}$ in the right hand side of (1.4), we obtain a linear equation which solution is non-negative and (1.6) gives N≥0; that this fixed point may work is a more involved issue, since we prove that there are not always global solutions, which requires functional spaces and this motives the *a priori* estimates that we derive at the end of this section.

Let us remark that the growth condition on the test function together with the assumption (1.7) imply that the term involving h(v,N) makes sense. By choosing test functions of the form ψ(t)ϕ(v), this formulation is equivalent to say that for all $\phi (v)\in C^{\infty }((-\infty ,V_{F}])$ such that $v\frac{\partial \phi }{\partial v}\in L^{\infty }((-\infty ,V_{F}))$, we have that

(2.2) $$\begin{array}{rl} & \frac{d}{dt}\int_{-\infty }^{V_{F}}\phi (v)p(v,t)\;dv\\ & \;=\int_{-\infty }^{V_{F}}[\frac{\partial \phi }{\partial v}h(v,N)+a\frac{\partial^{2}\phi }{\partial v^{2}}]p(v,t)\;dv+N(t)[\phi (V_{R})-\phi (V_{F})] \end{array}$$

holds in the distributional sense. It is trivial to check that weak solutions conserve the mass of the initial data by choosing ϕ=1 in (2.2), and thus,

(2.3) $$\int_{-\infty }^{V_{F}}p(v,t)\;dv=\int_{-\infty }^{V_{F}}p^{0}(v)\;dv=1.$$

The first result we show is that global-in-time weak solutions of (1.4)-(1.6) do not exist for all initial data in the case of an average-excitatory network. This result holds with less stringent hypotheses on the coefficients than in (1.7) with an analogous notion of weak solution as in Definition 2.1.

**Theorem 2.2** (Blow-up)

*Assume that the drift and diffusion coefficients satisfy*

(2.4) $$h(v,N)+v\geq bN\;\text{and}\;a(N)\geq a_{m}>0,$$

*for all*$-\infty <v\leq V_{F}$*and all*N≥0, *and let us consider the average*-*excitatory network where*b>0. *Choose*$\mu >\max(\frac{V_{F}}{a_{m}},\frac{1}{b})$. *If the initial data is concentrated enough around*$v=V_{F}$, *in the sense that*

$$\int_{-\infty }^{V_{F}}e^{\mu v}p^{0}(v)\;dv$$

*is close enough to*$e^{\mu V_{F}}$, *then there are no global*-*in*-*time weak solutions to* (1.4)-(1.6).

*Proof* We choose a multiplier $\phi (v)=e^{\mu v}$ with μ>0 and define the number

$$\lambda =\frac{\phi (V_{F})-\phi (V_{R})}{b\mu }>0$$

by hypotheses. For a weak solution according to (2.1), we find from (2.2) that

(2.5) $$\begin{array}{rl} & \frac{d}{dt}\int_{-\infty }^{V_{F}}\phi (v)p(v,t)\;dv\\ & \;\geq \mu \int_{-\infty }^{V_{F}}(bN(t)-v)\phi (v)p(v,t)\;dv\\ & \;+\mu^{2}a_{m}\int_{-\infty }^{V_{F}}\phi (v)p(v,t)\;dv-\lambda b\mu N(t)\\ & \;\geq \mu \int_{-\infty }^{V_{F}}\phi (v)p(v,t)\;dv\;[bN(t)+\mu a_{m}-V_{F}]-\lambda \mu bN(t), \end{array}$$

where (2.4) and the fact that $v\in (-\infty ,V_{F})$ was used. Let us now choose *μ* large enough such that $\mu a_{m}-V_{F}>0$ according to our hypotheses and denote

$$M_{\mu }(t)=\int_{-\infty }^{V_{F}}\phi (v)p(v,t)\;dv,$$

which satisfies

$$\frac{d}{dt}M_{\mu }(t)\geq b\mu N(t)[M_{\mu }(t)-\lambda ].$$

If initially $M_{\mu }(0)\geq \lambda$ and using Gronwall’s Lemma since N(t)≥0, we have that $M_{\mu }(t)\geq \lambda$, for all t≥0, and back to (2.5) we find

$$\frac{d}{dt}\int_{-\infty }^{V_{F}}\phi (v)p(v,t)\;dv\geq \mu (\mu a_{m}-V_{F})\int_{-\infty }^{V_{F}}\phi (v)p(v,t)\;dv$$

which in turn implies,

$$\int_{-\infty }^{V_{F}}\phi (v)p(v,t)\;dv\geq e^{\mu (\mu a_{m}-V_{F})t}\int_{-\infty }^{V_{F}}\phi (v)p^{0}(v)\;dv.$$

On the other hand, since p(v,t) is a probability density, see (2.3), and μ>0 then

$$\int_{-\infty }^{V_{F}}\phi (v)p(v,t)\;dv\leq e^{\mu V_{F}},$$

leading to a contradiction.

It remains to show that the set of initial data satisfying the size condition $M_{\mu }(0)\geq \lambda$ is not empty. To verify this, we can approximate as much as we want by smooth initial probability densities an initial Dirac mass at $V_{F}$ which gives the condition

$$e^{\mu V_{F}}\geq \lambda =\frac{e^{\mu V_{F}}-e^{\mu V_{R}}}{b\mu }\;\text{together with }\mu a_{m}>V_{F}.$$

This can be equivalently written as

$$\mu \geq \frac{1-e^{-\mu (V_{F}-V_{R})}}{b}\;\text{and}\;\mu >\frac{V_{F}}{a_{m}}.$$

Choosing $\mu >\max(\frac{1}{b},\frac{V_{F}}{a_{m}})$, these conditions are obviously fulfilled. □

 As usual for this type of blow-up result similar in spirit to the classical Keller-Segel model for chemotaxis [26,27], the proof only ensures that solutions for those initial data do not exist beyond a finite maximal time of existence. It does not characterize the nature of the first singularity which occurs. It implies that either the decay at infinity is false, although not probable, implying that the time evolution of probability densities ceases to be tight, or the function N(t) may become a singular measure in finite time instead of being an $L_{\mathrm{loc}}^{1}(R^{+})$ function. Actually, in the numerical computations shown in Section 5, we observe a blow-up in the value of the mean firing rate in finite time. To continue the solution will need a modification of the notion of solution introduced in Definition 2.1. It would be useful, since the firing rate does not become constant in time and consequently a possible interpretation is that synchronization occurs, a phenomena that is of interest to neuroscientists, see also the comments in the introduction and the conclusions.

Although in this paper the nature of blow-up is not mathematically identified, we devote the rest of the section to prove some *a priori* estimates which shed some light on this direction. To be more precise, our estimates indicate that this blow-up should not come from a loss of mass at v≈−∞, or a lack of fast decay rate because the second moment in *v* is controlled uniformly in blow-up situations. We obtain these *a priori* bounds with the help of appropriate choices of the test function *ϕ* in (2.1). Some of these choices are not allowed due to the growth at −∞ of the test functions. We will say that a weak solution is fast-decaying at −∞ if they are weak solutions in the sense of Definition 2.1 and the weak formulation in (2.2) holds for all test functions growing algebraically in *v*.

**Lemma 2.3** (*A priori* estimates)

*Assume* (1.7) *on the drift and diffusion coefficients and that*(p,N)*is a global*-*in*-*time solution of* (1.4)-(1.6) *in the sense of Definition *2.1 *fast decaying at* −∞, *then the following a priori estimates hold for all*T>0: 

(i) *If*$b\geq V_{F}-V_{R}$, *then*

$$\begin{array}{rcl} \int_{-\infty }^{V_{F}}(V_{F}-v)p(v,t)\;dv & \leq & \max(V_{F},\int_{-\infty }^{V_{F}}(V_{F}-v)p^{0}(v)\;dv),\\ (b-V_{F}+V_{R})\int_{0}^{T}N(t)\;dt & \leq & V_{F}T+\int_{-\infty }^{V_{F}}(V_{F}-v)p^{0}(v)\;dv. \end{array}$$

(ii) *If*$b<V_{F}-V_{R}$*then*

$$\int_{-\infty }^{V_{F}}(V_{F}-v)p(v,t)\;dv\geq \min(V_{F},\int_{-\infty }^{V_{F}}(V_{F}-v)p^{0}(v)\;dv).$$

*Moreover*, *if in additionais constant then*

(2.6) $$\int_{0}^{T}N(t)\;dt\leq (1+T)C(V_{F},V_{R},a,p^{0}).$$

*Proof* Using (1.7) together with our decay assumption at −∞, we may use the test function $\phi (v)=V_{F}-v\geq 0$. Then (2.2) gives

$$\begin{array}{rcl} & & \frac{d}{dt}\int_{-\infty }^{V_{F}}\phi (v)p(v,t)\;dv\\ & & \;=\int_{-\infty }^{V_{F}}[v-bN(t)]p(v,t)\;dv+N(t)(V_{F}-V_{R}). \end{array}$$

This is also written as

(2.7) $$\begin{array}{rl} & \frac{d}{dt}\int_{-\infty }^{V_{F}}\phi (v)p(v,t)\;dv+\int_{-\infty }^{V_{F}}\phi (v)p(v,t)\;dv\\ & \;=V_{F}-N(t)[b-(V_{F}-V_{R})]. \end{array}$$

To prove (i), we notice that with our condition on *b*, the term in N(t) is nonpositive and the first result follows from Gronwall’s inequality. The second result just follows after integration in time.

To prove (ii), we first use again (2.7) and, because the term in N(t) is nonnegative, we find the first result. To obtain the second estimate (2.6), given $\epsilon \in (0,(V_{F}-V_{R})/2)$, we can always choose a smooth truncation function $\phi_{\epsilon }(v)\in C^{2}$ such that

$$\phi_{\epsilon }(V_{F})=1,\;\phi_{\epsilon }(v)=0\;\text{for }v\leq V_{R},\;\phi_{\epsilon }^{\prime }(v)\geq 0,\;\phi_{\epsilon }^{\prime \prime }(v)\geq 0,$$

with $\phi_{\epsilon }^{\prime \prime }(v)=0$ outside the interval $\left(V_{R},V_{R}+\epsilon \right)$ such that

(2.8) $$\phi_{\epsilon }^{\prime }(v)\to \frac{1}{V_{F}-V_{R}}\;\text{for all }v\in (V_{R},V_{F}],\;\text{as }\epsilon \to 0,$$

and thus $\phi_{\epsilon }^{\prime \prime }\in L^{\infty }(-\infty ,V_{F})$ with size of order of $\epsilon^{-1}$. In other words, we have chosen a $C^{2}$ uniform approximation of the truncation ${\left(v-V_{R}\right)}_{+}/(V_{F}-V_{R})$ with $x_{+}=\max(x,0)$ obtained by integrating twice a smooth suitable approximation of the $\delta (V-V_{R})/(V_{F}-V_{R})$. Then, equation (2.2) gives

$$\begin{array}{rcl} & & \frac{d}{dt}\int_{V_{R}}^{V_{F}}\phi_{\epsilon }(v)p(v,t)\;dv+N(t)\\ & & \;=\int_{V_{R}}^{V_{F}}\phi_{\epsilon }^{\prime }(v)(-v+bN(t))p(v,t)\;dv+a\int_{V_{R}}^{V_{F}}\phi_{\epsilon }^{\prime \prime }(v)p(v,t)\;dv. \end{array}$$

Since $\phi_{\epsilon }^{\prime }(v)$ is positive and non-decreasing and using (2.8), we get for all $0<\tilde{\varepsilon }<1$ there exists $\epsilon_{0}$ small enough, such that for all $0<\epsilon <\epsilon_{0}$:

(2.9) $$\begin{array}{rl} & \left|\int_{V_{R}}^{V_{F}}\phi_{\epsilon }^{\prime }(v)(-v+bN(t))p(v,t)\;dv\right|\\ & \;\leq \phi_{\epsilon }^{\prime }(V_{F})(\max(|V_{F}|,|V_{R}|)+bN(t))\\ & \;\leq \frac{\left(1+\tilde{\varepsilon })(\max(|V_{F}|,|V_{R}|)+bN(t)\right)}{V_{F}-V_{R}}. \end{array}$$

Due to the hypotheses $b<V_{F}-V_{R}$, for any *γ* such that $0<\gamma <1-b/(V_{F}-V_{R})$, we can find $\tilde{\varepsilon }$ small enough such that

$$0<\gamma <1-\frac{(1+\tilde{\varepsilon })b}{V_{F}-V_{R}}.$$

Taking into account (2.8), (2.9), and that p(v,t) is a probability density, we have for $0<\epsilon <\epsilon_{0}$ small enough

$$\frac{d}{dt}\int_{V_{R}}^{V_{F}}\phi_{\epsilon }(v)p(v,t)\;dv+\gamma N(t)\leq \frac{\max(|V_{F}|,|V_{R}|)}{V_{F}-V_{R}}+a{∥\phi_{\epsilon }^{\prime \prime }∥}_{L^{\infty }(V_{R},V_{F})}.$$

Choosing now $\epsilon =\epsilon_{0}/2$ for instance, integration in time of the last inequality leads to the desired inequality (2.6). □

**Corollary 2.4***Under the assumptions of Lemma *2.3 *and assuming*$v^{2}p^{0}(v)\in L^{1}(-\infty ,V_{F})$*and*$0<b<V_{F}-V_{R}$, *then the following a priori estimates hold*: 

(i) *If additionallyais constant*, *for all*t≥0*we have*

$$\int_{-\infty }^{V_{F}}v^{2}p(v,t)\;dv\leq C(1+t).$$

(ii) *If additionally*$-b\min(V_{F},\int_{-\infty }^{V_{F}}(V_{F}-v)p^{0}(v)\;dv)+a_{1}+bV_{F}+\frac{V_{R}^{2}-V_{F}^{2}}{2}\leq 0$, *then*

$$\int_{-\infty }^{V_{F}}v^{2}p(v,t)\;dv\leq \max(a_{0},\int_{-\infty }^{V_{F}}v^{2}p^{0}(v,t)\;dv).$$

*Proof* We use again the weak formulation (2.1) with $\phi (v)=v^{2}/2$ as test function and get

$$\begin{array}{rl} & \frac{d}{dt}\int_{-\infty }^{V_{F}}\frac{v^{2}}{2}p(v,t)\;dv+\int_{-\infty }^{V_{F}}v^{2}p(v,t)\;dv\\ & \;=bN(t)\int_{-\infty }^{V_{F}}vp(v,t)\;dv+a(N(t))+N(t)\frac{V_{R}^{2}-V_{F}^{2}}{2}\\ & \;=bN(t)\int_{-\infty }^{V_{F}}(v-V_{F})p(v,t)\;dv+a(N(t))+N(t)[bV_{F}+\frac{V_{R}^{2}-V_{F}^{2}}{2}]\\ & \;\leq a_{0}+N(t)[-b\min(V_{F},\int_{-\infty }^{V_{F}}(V_{F}-v)p^{0}(v)\;dv)+a_{1}+bV_{F}+\frac{V_{R}^{2}-V_{F}^{2}}{2}] \end{array}$$

thanks to the first statement of Lemma 2.3(ii).

To prove (i), we just use the second statement of Lemma 2.3(ii) valid for *a* constant which tells us that the time integration of the right-hand side grows at most linearly in time and so does $\int_{-\infty }^{V_{F}}v^{2}p(v,t)\;dv$.

To prove (ii), we just use that the bracket is nonpositive and the results follows. □

## 3 Steady states

### 3.1 Generalities

This section is devoted to find all smooth stationary solutions of the problem (1.4)-(1.6) in the particular relevant case of a drift of the form $h(v)=V_{0}(N)-v$. Let us search for continuous stationary solutions *p* of (1.4) such that *p* is $C^{1}$ regular except possibly at $V=V_{R}$ where it is *Lipschitz*. Using the definition in (2.2), we are then allowed by a direct integration by parts in the second derivative term of *p* to deduce that *p* satisfies

(3.1) $$\frac{\partial }{\partial v}[(v-V_{0}(N))p(v)+a(N)\frac{\partial }{\partial v}p(v)+NH(v-V_{R})]=0$$

in the sense of distributions, with *H* being the Heaviside function, that is, H(u)=1 for u≥0 and H(u)=0 for u<0. Therefore, we conclude that

$$(v-V_{0}(N))p+a(N)\frac{\partial p}{\partial v}+NH(v-V_{R})=C.$$

The definition of *N* in (1.6) and the Dirichlet boundary condition (1.5) imply C=0 by evaluating this expression at $v=V_{F}$. Using again the boundary condition (1.5), $p(V_{F})=0$, we may finally integrate again and find that

$$p(v)=\frac{N}{a(N)}e^{-\frac{{\left(v-V_{0}(N)\right)}^{2}}{2a(N)}}\int_{v}^{V_{F}}e^{\frac{{\left(w-V_{0}(N)\right)}^{2}}{2a(N)}}H[w-V_{R}]\;dw$$

which can be rewritten, using the expression of the Heaviside function, as

(3.2) $$p(v)=\frac{N}{a(N)}e^{-\frac{{\left(v-V_{0}(N)\right)}^{2}}{2a(N)}}\int_{\max(v,V_{R})}^{V_{F}}e^{\frac{{\left(w-V_{0}(N)\right)}^{2}}{2a(N)}}\;dw.$$

Moreover, the firing rate in the stationary state *N* is determined by the normalization condition (2.3), or equivalently,

(3.3) $$\frac{a(N)}{N}=\int_{-\infty }^{V_{F}}[e^{-\frac{{\left(v-V_{0}(N)\right)}^{2}}{2a(N)}}\int_{\max(v,V_{R})}^{V_{F}}e^{\frac{{\left(w-V_{0}(N)\right)}^{2}}{2a(N)}}\;dw]\;dv.$$

Summarizing, all solutions *p* of the stationary problem (3.1), with the above referred regularity, are of the form given by the expression (3.2), where *N* is any positive solution of the implicit equation (3.3).

Let us first comment that in the linear case $V_{0}(N)=0$ and $a(N)=a_{0}>0$, we then get a unique stationary state $p_{\infty }$ given by the Dawson function

(3.4) $$p_{\infty }(v)=\frac{N_{\infty }}{a_{0}}e^{-\frac{v^{2}}{2a_{0}}}\int_{\max(v,V_{R})}^{V_{F}}e^{\frac{w^{2}}{2a_{0}}}\;dw,$$

with $N_{\infty }$ the normalizing constant to unit mass over the interval $\left(-\infty ,V_{F}\right]$.

 The rest of this section is devoted to find conditions on the parameters of the model clarifying the number of solutions to (3.3). With this aim, it is convenient to perform a change of variables, and use new notations

(3.5) $$z=\frac{v-V_{0}}{\sqrt{a}},\;u=\frac{w-V_{0}}{\sqrt{a}},\;w_{F}=\frac{V_{F}-V_{0}}{\sqrt{a}},\;w_{R}=\frac{V_{R}-V_{0}}{\sqrt{a}},$$

where the *N* dependency has been avoided to simplify notation. Then, as in [3], we can rewrite the previous integral (and thus the condition for a steady state) as

(3.6) $$\left\{ \begin{array}{l} \frac{1}{N}=I(N),\\ I(N):=\int_{-\infty }^{w_{F}}[e^{-\frac{z^{2}}{2}}\int_{\max(z,w_{R})}^{w_{F}}e^{\frac{u^{2}}{2}}\;du]\;dz. \end{array}\right.$$

Another alternative form of I(N) follows from the change of variables s=(z−u)/2 and $\tilde{s}=(z+u)/2$ to get

$$I(N)=2\int_{-\infty }^{0}\int_{w_{R}+s}^{w_{F}+s}e^{-2s\tilde{s}}\;d\tilde{s}\;ds=-\int_{-\infty }^{0}\frac{e^{-2s^{2}}}{s}(e^{-2sw_{F}}-e^{-2sw_{R}})\;ds,$$

and consequently,

(3.7) $$I(N)=\int_{0}^{\infty }\frac{e^{-s^{2}/2}}{s}(e^{sw_{F}}-e^{sw_{R}})\;ds.$$

### 3.2 Case of $a(N)=a_{0}$

We are now ready to state our main result on steady states.

**Theorem 3.1***Assume*h(v,N)=bN−v, $a(N)=a_{0}$*is constant and*$V_{0}=bN$. 

(i) *For*b<0*and*b>0*small enough there is a unique steady state to* (1.4)-(1.6).

(ii) *Under either the condition*

(3.8) $$0<b<V_{F}-V_{R},$$

*or the condition*

(3.9) $$0<2a_{0}b<{\left(V_{F}-V_{R}\right)}^{2}V_{R},$$

*then there exists at least one steady state solution to* (1.4)-(1.6).

(iii) *If both* (3.9) *and*$b>V_{F}-V_{R}$*hold*, *then there are at least two steady states to* (1.4)-(1.6).

(iv) *There is no steady state to* (1.4)-(1.6) *under the high connectivity condition*

(3.10) $$b>\max(2(V_{F}-V_{R}),2V_{F}I(0)).$$

**Remark 3.2***It is natural to relate the absence of steady state forblarge with blow*-*up of solutions*. *However*, *Theorem *2.2 *in Section *2*shows this is not the only possible cause since the blow*-*up can happen for initial data concentrated enough around*$V_{F}$*independently of the value of*b>0. *See also Section *5*for related numerical results*.

*Proof* Let us first study properties of the function I(N). To do that, we rewrite (3.7) as

$$I(N)=\int_{0}^{\infty }e^{-s^{2}/2}e^{-\frac{sbN}{\sqrt{a_{0}}}}\frac{e^{\frac{sV_{F}}{\sqrt{a_{0}}}}-e^{\frac{sV_{R}}{\sqrt{a_{0}}}}}{s}\;ds.$$

Taking the function $f(s)=e^{\frac{sV_{F}}{\sqrt{a_{0}}}}-e^{\frac{sV_{R}}{\sqrt{a_{0}}}}$ and Taylor expanding up to second order at s=0, we get $f(s)-f(0)-f^{\prime }(0)s=f^{\prime \prime }(\theta )s^{2}/2$ with f(0)=0, $f^{\prime }(0)=(V_{F}-V_{R})/\sqrt{a_{0}}$, and θ∈(0,s). It is easy to see that

$$|f^{\prime \prime }(\theta )|\leq \max(\frac{V_{F}^{2}}{2a_{0}}e^{\frac{\theta V_{F}}{\sqrt{a_{0}}}},\frac{V_{R}^{2}}{2a_{0}}e^{\frac{\theta V_{R}}{\sqrt{a_{0}}}}),$$

for all θ∈(0,s). By distinguishing the cases based on the signs of $V_{F}$ and $V_{R}$, this Taylor expansion implies that

(3.11) $$\begin{array}{rl} \left|\frac{e^{\frac{sV_{F}}{\sqrt{a_{0}}}}-e^{\frac{sV_{R}}{\sqrt{a_{0}}}}}{s}-\frac{V_{F}-V_{R}}{\sqrt{a_{0}}}\right| & \leq \frac{\max(V_{F}^{2},V_{R}^{2})}{2a_{0}}se^{\frac{s\max(|V_{F}|,|V_{R}|)}{\sqrt{a_{0}}}}\\ & :=C_{0}se^{\frac{s\max(|V_{F}|,|V_{R}|)}{\sqrt{a_{0}}}} \end{array}$$

for all s≥0. Then, a direct application of the dominated convergence theorem and continuity theorems of integrals with respect to parameters show that the function I(N) is continuous on *N* on [0,∞). Moreover, the function I(N) is $C^{\infty }$ on *N* since all their derivatives can be computed by differentiating under the integral sign by direct application of dominated convergence theorems and differentiation theorems of integrals with respect to parameters. In particular,

$$I^{\prime }(N)=-\frac{b}{\sqrt{a_{0}}}\int_{0}^{\infty }e^{-s^{2}/2}(e^{sw_{F}}-e^{sw_{R}})\;ds,$$

and for all integers k≥1,

$$I^{\left(k\right)}(N)={\left(-1\right)}^{k}{\left(\frac{b}{\sqrt{a_{0}}}\right)}^{k}\int_{0}^{\infty }e^{-s^{2}/2}s^{k-1}(e^{sw_{F}}-e^{sw_{R}})\;ds.$$

As a consequence, we deduce: 

1. Case b<0: I(N) is an increasing strictly convex function and thus

$$\lim_{N\to \infty }I(N)=\infty .$$

2. Case b>0: I(N) is a decreasing convex function. Also, it is obvious from the previous expansion (3.11) and dominated convergence theorem that

$$\lim_{N\to \infty }I(N)=0.$$

It is also useful to keep in mind that, thanks to the form of I(N) in (3.6),

(3.12) $$\begin{array}{rl} I(0) & \leq \sqrt{2\pi }[w_{F}(0)-w_{R}(0)]e^{\max(w_{R}^{2}(0),w_{F}^{2}(0))/2}\\ & =\sqrt{2\pi }\frac{\left(V_{F}-V_{R}\right)}{\sqrt{a_{0}}}\exp\{\frac{\max(V_{R}^{2},V_{F}^{2})}{2a_{0}}\}<\infty . \end{array}$$

Now, let us show that for b>0, we have

(3.13) $$\lim_{N\to \infty }NI(N)=\frac{V_{F}-V_{R}}{b}.$$

Using (3.11), we deduce

$$\begin{array}{rcl} & & \left|NI(N)-N\frac{V_{F}-V_{R}}{\sqrt{a_{0}}}\int_{0}^{\infty }e^{-s^{2}/2}e^{-\frac{sbN}{\sqrt{a_{0}}}}\;ds\right|\\ & & \;\leq C_{0}N\int_{0}^{\infty }se^{-s^{2}/2}e^{-\frac{sbN}{\sqrt{a_{0}}}}e^{\frac{s\max(|V_{F}|,|V_{R}|)}{\sqrt{a_{0}}}}\;ds. \end{array}$$

A direct application of dominated convergence theorem shows that the right hand side converges to 0 as N→∞ since $sN\exp(-\frac{sbN}{\sqrt{a_{0}}})$ is a bounded function uniform in *N* and *s*. Thus, the computation of the limit is reduced to show

(3.14) $$\lim_{N\to \infty }N\int_{0}^{\infty }e^{-s^{2}/2-\frac{sbN}{\sqrt{a_{0}}}}\;ds=\frac{\sqrt{a_{0}}}{b}.$$

With this aim, we rewrite the integral in terms of the complementary error function defined as

$$\mathrm{erfc}(x):=\frac{2}{\sqrt{\pi }}\int_{x}^{\infty }e^{-t^{2}}\;dt,$$

and then

$$\int_{0}^{\infty }e^{-s^{2}/2-\frac{sbN}{\sqrt{a_{0}}}}\;ds=e^{\frac{b^{2}N^{2}}{2a_{0}}}\int_{0}^{\infty }e^{-{\left(\frac{s}{\sqrt{2}}+\frac{bN}{\sqrt{2a_{0}}}\right)}^{2}}\;ds=\frac{\sqrt{\pi }}{\sqrt{2}}e^{\frac{b^{2}N^{2}}{2a_{0}}}\mathrm{erfc}(\frac{bN}{\sqrt{2a_{0}}}).$$

Finally, we can obtain the limit (3.14) using L’Hôpital’s rule

$$\begin{array}{rcl} \lim_{N\to \infty }N\int_{0}^{\infty }e^{-s^{2}/2-\frac{sbN}{\sqrt{a_{0}}}}\;ds & = & \frac{\sqrt{\pi }}{\sqrt{2}}\lim_{N\to \infty }\frac{\mathrm{erfc}(\frac{bN}{\sqrt{2a_{0}}})}{\frac{e^{-\frac{b^{2}N^{2}}{2a_{0}}}}{N}}\\ & = & \sqrt{2}\lim_{N\to \infty }\frac{-\frac{b}{\sqrt{2a_{0}}}e^{-\frac{b^{2}N^{2}}{2a_{0}}}}{-\frac{b^{2}}{a_{0}}e^{-\frac{b^{2}N^{2}}{2a_{0}}}-\frac{1}{N^{2}}e^{-\frac{b^{2}N^{2}}{2a_{0}}}}=\frac{\sqrt{a_{0}}}{b}. \end{array}$$

With this analysis of the function I(N) we can now proof each of the statements of Theorem 3.1:

*Proof of (i)* Let us start with the case b<0. Here, the function I(N) is increasing, starting at I(0)<∞ due to (3.12) and such that

$$\lim_{N\to \infty }I(N)=\infty .$$

Therefore, it crosses to the function 1/N at a single point.

Now, for the case b>0 small, we first remark that similar dominated convergence arguments as above show that both I(N) and $I^{\prime }(N)$ are smooth functions of *b*. Moreover, it is simple to realize that I(N) is a decreasing function of the parameter *b*. Now, choosing $0<b\leq b_{*}<(V_{F}-V_{R})/2$, then $I(N)\geq I_{*}(N)$ for all N≥0 where $I_{*}(N)$ denotes the function associated to the parameter $b_{*}$. Using the limit (3.13), we can now infer the existence of $N_{*}>0$ depending only on $b_{*}$ such that

$$NI(N)\geq NI_{*}(N)>\frac{V_{F}-V_{R}}{2b_{*}}>1$$

for all $N\geq N_{*}$. Therefore, by continuity of NI(N) there are solutions to NI(N)=1 and all possible crossings of I(N) and 1/N are on the interval $\left[0,N_{*}\right]$. We observe that both I(N) and $I^{\prime }(N)$ converge towards the constant function I(0)>0 and to 0 respectively, uniformly in the interval $\left[0,N_{*}\right]$ as b→0. Therefore, for *b* small NI(N) is strictly increasing on the interval $\left[0,N_{*}\right]$ and there is a unique solution to NI(N)=1. □

Proof of (ii)

**Case of (3.8)** The claim that there are solutions to NI(N)=1 for $0<b<V_{F}-V_{R}$ is a direct consequence of the continuity of I(N), (3.12) and (3.13).

**Case of (3.9)** We are going to prove that I(N)≥1/N for $\frac{2a_{0}}{{\left(V_{R}-V_{F}\right)}^{2}}<N<\frac{V_{R}}{b}$, which concludes the existence of a steady state since I(0)<∞ due to (3.12) implies that I(N)<1/N for small *N*. Condition (3.9) only asserts that this interval for *N* is not empty. To do so, we show that

$$I(N)\geq \frac{{\left(V_{R}-V_{F}\right)}^{2}}{2a_{0}}\;\text{for }N\in [0,\frac{V_{R}}{b}]$$

which obviously concludes the desired inequality I(N)≥1/N for the interval of *N* under consideration.

The condition $\frac{V_{R}}{b}>N$ is equivalent to $w_{R}>0$, therefore, using (3.5) and the expression for I(N) in (3.6), we deduce

$$I(N)\geq \int_{w_{R}}^{w_{F}}[e^{-\frac{z^{2}}{2}}\int_{\max(z,w_{R})}^{w_{F}}e^{\frac{u^{2}}{2}}\;du]\;dz\geq \int_{w_{R}}^{w_{F}}[e^{-\frac{z^{2}}{2}}\int_{z}^{w_{F}}e^{\frac{u^{2}}{2}}\;du]\;dz.$$

Since z>0 and $e^{\frac{u^{2}}{2}}$ is an increasing function for u>0, then $e^{\frac{u^{2}}{2}}\geq e^{\frac{z^{2}}{2}}$ on $\left[z,w_{F}\right]$, and we conclude 

*Proof of (iii)* Under the condition (3.9), we have shown in the previous point the existence of an interval where I(N)>I/N. On one hand, I(0)<∞ in (3.12) implies that I(N)<I/N for *N* small and the condition $b>V_{F}-V_{R}$ implies that I(N)<I/N for *N* large enough due to the limit (3.13), thus there are at least two crossings between I(N) and 1/N. □

*Proof of (iv)* Under assumption (3.10) for *b*, it is easy to check that the following inequalities hold

(3.15) $$I(0)<1/N\;\text{for }N\leq 2V_{F}/b$$

and

(3.16) $$\frac{V_{F}-V_{R}}{bN-V_{F}}<\frac{1}{N}\;\text{for }N>2V_{F}/b.$$

We consider *N* such that $N>V_{F}/b$, this means that $w_{F}<0$. We use the formula (3.7) for I(N) and write the inequalities

$$\begin{array}{rl} I(N) & <(w_{F}-w_{R})\int_{0}^{\infty }e^{-s^{2}/2}e^{sw_{F}}=(w_{F}-w_{R})e^{w_{F}^{2}/2}\int_{0}^{\infty }e^{-{\left(s-w_{F}\right)}^{2}/2}\\ & =(w_{F}-w_{R})e^{w_{F}^{2}/2}\int_{-w_{F}}^{\infty }e^{-s^{2}/2}\leq (w_{F}-w_{R})e^{w_{F}^{2}/2}\int_{-w_{F}}^{\infty }\frac{s}{\left|w_{F}\right|}e^{-s^{2}/2}\\ & =\frac{V_{F}-V_{R}}{{\sqrt{a}}_{0}|w_{F}|} \end{array}$$

where the mean-value theorem and $w_{F}<0$ were used. Then, we conclude that

$$I(N)<\frac{V_{F}-V_{R}}{{\sqrt{a}}_{0}|w_{F}|}=\frac{V_{F}-V_{R}}{bN-V_{F}},\;\text{for }N>V_{F}/b.$$

Therefore, using Inequality (3.16):

$$I(N)<\frac{1}{N},\;\text{for }N>2V_{F}/b$$

and due to the fact that *I* is decreasing and Inequality (3.15), we have I(N)<I(0)<1/N, for $N\leq 2V_{F}/b$. In this way, we have shown that for all *N*, I(N)<1/N and consequently there is no steady state. □

**Fig. 1:**
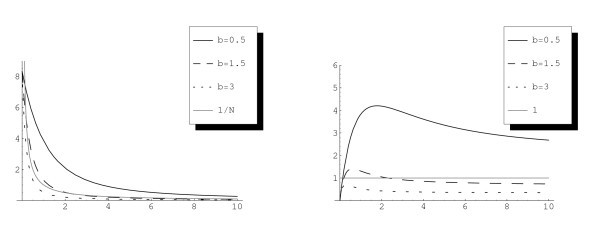
For several values of *b*, the function I(N) in (3.6) is plotted against the function 1/N (left figure) and the function NI(N) against the constant function 1 (right figure). Here a≡1, $V_{R}=1$, $V_{F}=2$.

**Remark 3.3***The functions*I(N)*and*1/N*are depicted in Figure *1*for the case*$V_{0}(N)=bN$*and*$a(N)=a_{0}$*illustrating the main result*: *steady states exist for smallband do not exist for largebwhile there is an intermediate range of existence of two stationary states*. *The numerical plots of the function*NI(N)*might indicate that there are only three possibilities*: *one stationary state*, *two stationary states and no stationary state*. *However*, *we are not able to prove or disprove the uniqueness of a maximum for the function*NI(N)*eventually giving this sharp result*. 

**Remark 3.4***The condition* (3.9) *is not optimal and it can be improved by using one more term in the series expansion of the exponentials inside the integral of the expression of*I(N)*in* (3.7). *More precisely*, *if*$w_{F}>w_{R}>0$, *we use*

$$e^{sw_{F}}-e^{sw_{R}}=\sum_{n=0}^{\infty }\frac{s^{n}}{n!}(w_{F}^{n}-w_{R}^{n})\geq \sum_{n=0}^{2}\frac{s^{n}}{n!}(w_{F}^{n}-w_{R}^{n}).$$

*In this way*, *we get*

$$\begin{array}{rcl} I(N) & \geq & \int_{0}^{\infty }e^{-s^{2}/2}(\frac{V_{F}-V_{R}}{\sqrt{a_{0}}}+\frac{1}{2}(w_{F}^{2}-w_{R}^{2})s)\;ds\\ & \geq & \frac{\left(V_{F}-V_{R})(\sqrt{2\pi a_{0}}+(V_{F}-V_{R})\right)}{2a_{0}}, \end{array}$$

since

$$\int_{0}^{\infty }e^{-s^{2}/2}\;ds=\sqrt{\frac{\pi }{2}},\;\int_{0}^{\infty }e^{-s^{2}/2}s\;ds=1\;\text{and}\;V_{0}<V_{R}.$$

*Then*, *condition* (3.9) *can be improved to*

$$2a_{0}b<V_{R}(V_{F}-V_{R})(\sqrt{2\pi a_{0}}+(V_{F}-V_{R})).$$

*Of course*, *this last inequality is not optimal either for the same reason as before*.

### 3.3 Case of $a(N)=a_{0}+a_{1}N$

We now treat the case of $a(N)=a_{0}+a_{1}N$, with $a_{0},a_{1}>0$ with b>0. Proceeding as above we can obtain from (3.7) the expression of its derivative

(3.17) $$\begin{array}{rl} I^{\prime }(N)= & -\frac{d}{dN}[\frac{V_{0}(N)}{\sqrt{a(N)}}](I_{1}(N)-I_{2}(N))\\ & +\frac{d}{dN}(\frac{1}{\sqrt{a(N)}})(V_{F}I_{1}(N)-V_{R}I_{2}(N)), \end{array}$$

where

$$I_{1}(N)=\int_{0}^{\infty }e^{-s^{2}/2}e^{sw_{F}}\;ds\;\text{and}\;I_{2}(N)=\int_{0}^{\infty }e^{-s^{2}/2}e^{sw_{R}}\;ds.$$

Therefore I(N) is decreasing since

$$\begin{array}{rcl} \frac{d}{dN}[\frac{V_{0}(N)}{\sqrt{a(N)}}] & = & \frac{2ba_{0}+ba_{1}N}{2{\left(a_{0}+a_{1}N\right)}^{3/2}}>0\;\text{and}\;\\ \frac{d}{dN}(\frac{1}{\sqrt{a(N)}}) & = & -\frac{a_{1}}{{\left(a_{0}+a_{1}N\right)}^{3/2}}<0. \end{array}$$

Moreover, we can check that the computation of the limit (3.13) still holds. Actually, we have

$$\begin{array}{rl} \lim_{N\to \infty }\frac{NI(N)}{V_{F}-V_{R}} & =\lim_{N\to \infty }\frac{N}{\sqrt{a}}\int_{0}^{\infty }e^{-s^{2}/2}e^{-sbN/\sqrt{a}}\;ds=\lim_{N\to \infty }\sqrt{\pi }\frac{\mathrm{erfc}(\frac{bN}{\sqrt{2a}})}{\frac{e^{-\frac{b^{2}N^{2}}{2a}}}{\frac{N}{\sqrt{2a}}}}\\ & =\lim_{\alpha \to \infty }\sqrt{\pi }\frac{\mathrm{erfc}(b\alpha )}{\frac{e^{-b^{2}\alpha^{2}}}{\alpha }}=\lim_{\alpha \to \infty }\sqrt{\pi }\frac{-\frac{2}{\sqrt{\pi }}be^{-b^{2}\alpha^{2}}}{\frac{-2b^{2}\alpha^{2}e^{-b^{2}\alpha^{2}}-e^{-b^{2}\alpha^{2}}}{\alpha^{2}}}=\frac{1}{b}, \end{array}$$

where we have used the change $\alpha =\frac{N}{\sqrt{2a}}$ and L’Hôpital’s rule. In the case b<0, we can observe again by the same proof as before that I(N)→∞ when N→∞, and thus, by continuity there is at least one solution to NI(N)=1. Nevertheless, it seems difficult to clarify perfectly the number of solutions due to the competing monotone functions in (3.17).

The generalization of part of Theorem 3.1 is contained in the following result. We will skip its proof since it essentially follows the same steps as before with the new ingredients just mentioned.

**Corollary 3.5***Assume*h(v,N)=bN−v, $a(N)=a_{0}+a_{1}N$*with*$a_{0},a_{1}>0$. 

(i) *Under either the condition*$b<V_{F}-V_{R}$, *or the conditions*b>0*and*$2a_{0}b+2a_{1}V_{R}<{\left(V_{F}-V_{R}\right)}^{2}V_{R}$, *then there exists at least one steady state solution to* (1.4)-(1.6).

(ii) *If both*$2a_{0}b+2a_{1}V_{R}<{\left(V_{F}-V_{R}\right)}^{2}V_{R}$*and*$b>V_{F}-V_{R}$*hold*, *then there are at least two steady states to* (1.4)-(1.6).

(iii) *There is no steady state to* (1.4)-(1.6) *for*$b>\max(2(V_{F}-V_{R}),2V_{F}I(0))$.

**Fig. 2:**
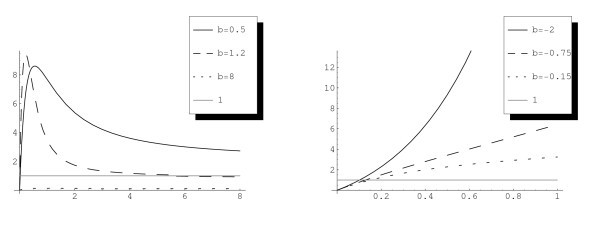
Left figure: The function NI(N) against the constant 1 when a(N) is linear. For b=0.5 we have considered a(N)=0.5+N/8, for b=1.2: a(N)=0.4+N/100 and for b=8: a(N)=6+N/100. Right figure: The function NI(N) against the constant 1 with b<0 and a(N)=1+N. Here $V_{R}=1$; $V_{F}=2$.

These behaviours are depicted in Figure 2. Let us point out that if *a* is linear and b<0, I(N) has not to be strictly increasing as in the constant diffusion case and it may have a minimum for N>0. 

At this point, a natural question is what happens with the stability of these steady states. In the next section we study it in the linear case, when the model presents only one steady state. An extension of the same techniques, entropy methods, to the nonlinear case is not straightforward at all. However, the results obtained in the linear case let us expect that for small connectivity parameter *b* the only steady state could be stable. On the other hand, numerical results presented in Section 5 give some numerical evidence of the stability/instability in different situations described by this model: only one steady state or two steady states, see that section for details.

## 4 Linear equation and relaxation

 We study specifically the case of a linear equation that is b=0 and $a(N)=a_{0}$, that is,

(4.1) $$\left\{ \begin{array}{ll} \frac{\partial p(v,t)}{\partial t}-\frac{\partial }{\partial v}[vp(v,t)]-a_{0}\frac{\partial^{2}}{\partial v^{2}}p(v,t)=\delta (v-V_{R})N(t), & v\leq V_{F},\\ p(V_{F},t)=0,\;N(t):=-a_{0}\frac{\partial }{\partial v}p(V_{F},t)\geq 0,\;a_{0}>0,\\ p(v,0)=p^{0}(v)\geq 0,\;\int_{-\infty }^{V_{F}}p^{0}(v)\;dv=1. \end{array}\right.$$

For later purposes, we remind that the steady state $p_{\infty }(v)$ given in (3.4) satisfies, for the case at hand, the equation

(4.2) $$\left\{ \begin{array}{ll} -\frac{\partial }{\partial v}[vp_{\infty }(v)]-a_{0}\frac{\partial^{2}}{\partial v^{2}}p_{\infty }(v)=\delta (v-V_{R})N_{\infty }, & v\leq V_{F},\\ p_{\infty }(V_{F})=0,\;N_{\infty }:=-a_{0}\frac{\partial }{\partial v}p_{\infty }(V_{F})\geq 0,\\ \int_{-\infty }^{V_{F}}p_{\infty }(v)\;dv=1. \end{array}\right.$$

We will assume in this section that the initial data satisfies, for some $C^{0}>0$

(4.3) $$p^{0}(v)\leq C^{0}p_{\infty }(v).$$

Then, we take for granted that solutions of the linear problem exist, with the regularity needed in each result below and such that for all t≥0

(4.4) $$p(v,t)\leq C^{0}p_{\infty }(v).$$

The estimate (4.4) follows *a posteriori* from the relative entropy inequality that we state below, see more comments at the end of this section. This is an indication that the hypothesis for the initial data (4.3) will easily be propagated in time giving (4.4) with a well-posedness theory of classical fast-decaying solutions at hand. These solutions to (4.1) and (4.2) might be obtained by the method developed in [28] and will be analysed elsewhere.

We prove that the solutions to (4.1) converge in large times to the unique steady state $p_{\infty }(v)$.

**Theorem 4.1** (Exponential decay)

*Fast*-*decaying solutions to the equation* (4.1) *verifying* (4.4) *satisfy*

$$\int_{-\infty }^{V_{F}}p_{\infty }(v){\left(\frac{p(v,t)-p_{\infty }(v)}{p_{\infty }(v)}\right)}^{2}\;dv\leq e^{-2a_{0}\nu t}\int_{-\infty }^{V_{F}}p_{\infty }(v){\left(\frac{p^{0}(v)-p_{\infty }(v)}{p_{\infty }(v)}\right)}^{2}\;dv.$$

This result shows that no synchronization of neuronal activity can be expected when the network is not connected, since solutions tend to produce a constant firing rate, a very intuitive conclusion. Because the rate of decay is exponential, we also expect that small connectivity cannot create synchronization either, again an intuitive conclusion proved rigorously for the elapsed time structured model in [22]. Also the proof shows that two relaxation processes are involved in this effect: dissipation by the diffusion term and dissipation by the firing term. These relaxation effects are stated in the following theorem which also gives the natural bounds for the solutions to equation (4.1) (choosing $G(u)=u^{2}$ gives the natural energy space of the system, a weighted $L^{2}$ space).

**Theorem 4.2** (Relative entropy inequality)

*Fast*-*decaying solutions to equation* (4.1) *verifying* (4.4) *satisfy*, *for any smooth convex function*$G:R^{+}⟶R$, *the inequality*

(4.5) $$\frac{d}{dt}\int_{-\infty }^{V_{F}}p_{\infty }(v)G(\frac{p(v,t)}{p_{\infty }(v)})\;dv=-D_{G}[p](t)\leq 0,$$

*with*

$$\begin{array}{rl} D_{G}[p](t)= & N_{\infty }[G(\frac{N(t)}{N_{\infty }})-G(\frac{p(v,t)}{p_{\infty }(v)})-(\frac{N(t)}{N_{\infty }}-\frac{p(v,t)}{p_{\infty }(v)})G^{\prime }(\frac{p(v,t)}{p_{\infty }(v)})]{|}_{V_{R}}\\ & +a_{0}\int_{-\infty }^{V_{F}}p_{\infty }(v)G^{\prime \prime }(\frac{p(v,t)}{p_{\infty }(v)}){\left[\frac{\partial }{\partial v}(\frac{p(v,t)}{p_{\infty }(v)})\right]}^{2}\;dv\geq 0. \end{array}$$

*Proof of Theorem 4.1* The proof is standard in Fokker-Planck theory and follows by applying the relative entropy inequality (4.5) with $G(x)={\left(x-1\right)}^{2}$. Then, we obtain

(4.6) $$\frac{d}{dt}\int_{-\infty }^{V_{F}}p_{\infty }(v){\left(\frac{p(v,t)}{p_{\infty }(v)}-1\right)}^{2}\;dv\leq -2a_{0}\int_{-\infty }^{V_{F}}p_{\infty }(v){\left[\frac{\partial }{\partial v}(\frac{p(v,t)}{p_{\infty }(v)}-1)\right]}^{2}\;dv.$$

To proceed further, we need an additional technical ingredient that we state and whose proof is postponed to an Appendix.

**Proposition 4.3***There exists*ν>0*such that*

$$\nu \int_{-\infty }^{V_{F}}p_{\infty }(v){\left(\frac{q(v)}{p_{\infty }(v)}\right)}^{2}\;dv\leq \int_{-\infty }^{V_{F}}p_{\infty }(v){\left[\frac{\partial }{\partial v}(\frac{q(v)}{p_{\infty }(v)})\right]}^{2}\;dv$$

*for all functionsqsuch*$\frac{q}{p_{\infty }}\in H^{1}(p_{\infty }(v)\;dv)$*and*$\int_{-\infty }^{V_{F}}q(v)\;dv=0$.

Poincaré’s-like inequality in Proposition 4.3 applied to $q=p-p_{\infty }$ bounds the right hand side on (4.6)

$$\frac{d}{dt}\int_{-\infty }^{V_{F}}p_{\infty }(v){\left(\frac{p(v,t)}{p_{\infty }(v)}-1\right)}^{2}\;dv\leq -2a_{0}\nu \int_{-\infty }^{V_{F}}p_{\infty }(v){\left(\frac{p(v,t)}{p_{\infty }(v)}-1\right)}^{2}\;dv.$$

Finally, the Gronwall lemma directly gives the result.  □

To show Theorem 4.2, which was used in the proof of Theorem 4.1, we need the following preliminary computations.

**Lemma 4.4***Givenpa fast*-*decaying solution of* (4.1) *verifying* (4.4), $p_{\infty }$*given by* (3.4) *andGa*$C^{2}$*convex function*, *then the following relations hold*: (4.7)

(4.8) $$\begin{array}{rl} & \frac{\partial }{\partial t}G(\frac{p}{p_{\infty }})-(v+\frac{2a_{0}}{p_{\infty }}\frac{\partial }{\partial v}p_{\infty })\frac{\partial }{\partial v}G(\frac{p}{p_{\infty }})-a_{0}\frac{\partial^{2}}{\partial v^{2}}G(\frac{p}{p_{\infty }})\\ & \;=-a_{0}G^{\prime \prime }(\frac{p}{p_{\infty }}){\left(\frac{\partial }{\partial v}\frac{p}{p_{\infty }}\right)}^{2}+\frac{N_{\infty }}{p_{\infty }}\delta (v-V_{R})(\frac{N}{N_{\infty }}-\frac{p}{p_{\infty }})G^{\prime }(\frac{p}{p_{\infty }}), \end{array}$$

(4.9)

*Proof* Since $\frac{\partial }{\partial v}(\frac{p}{p_{\infty }})=\frac{1}{p_{\infty }}\frac{\partial p}{\partial v}-\frac{p}{p_{\infty }^{2}}\frac{\partial p_{\infty }}{\partial v}$ we obtain

$$\frac{\partial p}{\partial v}=p_{\infty }\frac{\partial }{\partial v}(\frac{p}{p_{\infty }})+\frac{p}{p_{\infty }}\frac{\partial p_{\infty }}{\partial v}$$

and

$$\frac{\partial^{2}p}{\partial v^{2}}=p_{\infty }\frac{\partial^{2}}{\partial v^{2}}(\frac{p}{p_{\infty }})+2\frac{\partial }{\partial v}(\frac{p}{p_{\infty }})\frac{\partial p_{\infty }}{\partial v}+\frac{p}{p_{\infty }}\frac{\partial^{2}p_{\infty }}{\partial v^{2}}.$$

Using these two expressions in

$$\begin{array}{rcl} \frac{\partial }{\partial t}(\frac{p}{p_{\infty }}) & = & \frac{1}{p_{\infty }}\frac{\partial p}{\partial t}\\ & = & \frac{1}{p_{\infty }}\{\delta (v=V_{R})N(t)+\frac{\partial }{\partial v}[vp(v,t)]+a_{0}\frac{\partial^{2}}{\partial v^{2}}p(v,t)\} \end{array}$$

we obtain (4.7).

Equation (4.8) is a consequence of Equation (4.7) and the following expressions for the partial derivatives of $G(\frac{p}{p_{\infty }})$:

$$\frac{\partial }{\partial t}G(\frac{p}{p_{\infty }})=G^{\prime }(\frac{p}{p_{\infty }})\frac{\partial }{\partial t}(\frac{p}{p_{\infty }}),\;\frac{\partial }{\partial v}G(\frac{p}{p_{\infty }})=G^{\prime }(\frac{p}{p_{\infty }})\frac{\partial }{\partial v}(\frac{p}{p_{\infty }})$$

and

$$\frac{\partial^{2}}{\partial v^{2}}G(\frac{p}{p_{\infty }})=G^{\prime \prime }(\frac{p}{p_{\infty }}){\left(\frac{\partial }{\partial v}(\frac{p}{p_{\infty }})\right)}^{2}+G^{\prime }(\frac{p}{p_{\infty }})\frac{\partial^{2}}{\partial v^{2}}(\frac{p}{p_{\infty }}).$$

Finally, Equation (4.9) is obtained using Equation (4.8) and the fact that $p_{\infty }$ is solution of (4.2). □

*Proof of Theorem 4.2* We integrate from −∞ to $V_{F}-\alpha$ in (4.9) and let *α* tend to 0^+^ and use L’Hôpital’s rule

(4.10) $$\lim_{v\to V_{F}}\frac{p(v,t)}{p_{\infty }(v)}=\lim_{v\to V_{F}}\frac{\frac{\partial p}{\partial v}(v,t)}{\frac{\partial p_{\infty }}{\partial v}(v)}=\frac{N(t)}{N_{\infty }}.$$

Since $p(v,t)\leq C^{0}p_{\infty }(v)$, then

$$\begin{array}{rl} & \frac{d}{dt}\int_{-\infty }^{V_{F}}p_{\infty }G(\frac{p}{p_{\infty }})\;dv-a_{0}\frac{\partial }{\partial v}[p_{\infty }G(\frac{p}{p_{\infty }})]{|}_{V_{F}}\\ & \;=-a_{0}\int_{-\infty }^{V_{F}}p_{\infty }G^{\prime \prime }(\frac{p}{p_{\infty }}){\left(\frac{\partial }{\partial v}\frac{p}{p_{\infty }}\right)}^{2}\;dv\\ & \;+N_{\infty }[(\frac{N}{N_{\infty }}-\frac{p}{p_{\infty }})G^{\prime }(\frac{p}{p_{\infty }})+G(\frac{p}{p_{\infty }})]{|}_{V_{R}}. \end{array}$$

The Dirichlet boundary condition (1.5) implies that

$$-a_{0}\frac{\partial }{\partial v}[p_{\infty }G(\frac{p}{p_{\infty }})]{|}_{V_{F}}=-a_{0}\frac{\partial p_{\infty }}{\partial v}G(\frac{p}{p_{\infty }}){|}_{V_{F}}=N_{\infty }G(\frac{N(t)}{N_{\infty }}),$$

where we used that

$$\begin{array}{rcl} p_{\infty }\frac{\partial }{\partial v}G(\frac{p}{p_{\infty }}){|}_{V_{F}} & = & p_{\infty }G^{\prime }(\frac{p}{p_{\infty }})\frac{-Np_{\infty }+N_{\infty }p}{p_{\infty }^{2}a_{0}}{|}_{V_{F}}\\ & = & G^{\prime }(\frac{p}{p_{\infty }})(\frac{-N}{a_{0}}+\frac{N_{\infty }}{a_{0}}\frac{p}{p_{\infty }}){|}_{V_{F}}=0, \end{array}$$

due to (4.10). Collecting all terms leads to the desired inequality. □

 Let us finally remark that as a usual consequence of the General Relative Entropy principle (GRE) [29], the estimate (4.4) follows by choosing the convex function $G(x)={\left(x-C^{0}\right)}_{+}^{4}$. This shows that the bound (4.4) can be proved using (4.3) together with a well-posedness theory of classical fast-decaying at −∞ solutions to (4.1).

## 5 Numerical results

 We consider two different explicit methods to simulate the NNLIF (1.4). The first one is based on standard shock-capturing methods for the advection term and standard centered finite differences for the second-order term. More precisely, the first order term is approximated by finite difference WENO-schemes [30].

 The second numerical method is based on another finite difference scheme for the Fokker-Planck equation proposed in the literature called the Chang-Cooper method [31]. This method was also used in [20] for a computational neuroscience model with variable voltage and conductance. In order to use this method, the first step is to rewrite the Fokker-Planck equation (1.4) in terms of the Maxwellian $M(v)=e^{-\frac{{\left(v-bN\right)}^{2}}{2a(N)}}$ as follows,

$$\frac{\partial p}{\partial t}(v,t)-a(N(t))\frac{\partial }{\partial v}[M(v,t)\frac{\partial }{\partial v}(\frac{p(v,t)}{M(v,t)})]=N(t)\delta (v-V_{R}).$$

Then, the Chang-Cooper method performs a kind of *θ*-finite difference approximation of p/M, see [31] for details. The Chang-Cooper method presents difficulties when the firing rate becomes large and the diffusion coefficient a(N) is constant. More precisely, given $a(N)=a_{0}$ and b>0, if *N* is large, the drift of the Maxwellian, in terms of which is rewritten the Fokker-Planck equation, practically vanishes on the interval $\left(-\infty ,V_{F}\right]$ and this particular Chang-Cooper method is not suitable. Whenever a(N) is not constant, this problem disappears.

 Summarizing, we consider two different schemes for our simulations: the first one is based on WENO-finite differences as described above, and the second one by means of the cited Chang-Cooper method. In both cases the evolution on time is performed with a TVD Runge-Kutta scheme. In Section 2 of [20] these schemes are explained in details and we refer to [30,31] for a deeper analysis of them.

In our simulations we consider a uniform mesh in *v*, for $v\in [V_{\min},V_{F}]$. The value $V_{\min}$ (less than $V_{R}$) is adjusted in the numerical experiments to fulfill that $p(V_{\min},t)\approx 0$, while $V_{F}$ is fixed to 2 and $V_{R}=1$. As initial data we have taken two different types of functions: 

• Maxwellians:

(5.1) $$p^{0}(v)=\frac{1}{\sqrt{2\pi }\sigma_{0}}e^{-\frac{{\left(v-v_{0}\right)}^{2}}{2\sigma_{0}^{2}}},$$

where the mean $v_{0}$ and the variance $\sigma_{0}^{2}$ are chosen according to the analyzed phenomenon.

• Stationary Profiles (3.2) given by

$$p(v)=\frac{N}{a(N)}e^{-\frac{{\left(v-V_{0}(N)\right)}^{2}}{2a(N)}}\int_{\max(v,V_{R})}^{V_{F}}e^{\frac{{\left(w-V_{0}(N)\right)}^{2}}{2a(N)}}\;dw,$$

with *N* an approximate value of the stationary firing rate. We typically consider this kind of initial data to analyze local stability of steady states.

*Steady states. -* As we show in Section 3, for *b* positive there is a range of values for which there are either one or two or no steady states. With our simulations we can observe all the cases represented in Figures 1 and 2.

**Fig. 3:**
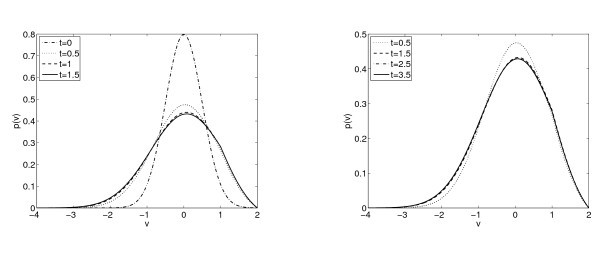
Distribution functions p(v,t) for b=0.5 and a≡1 at differents times.

**Fig. 4:**
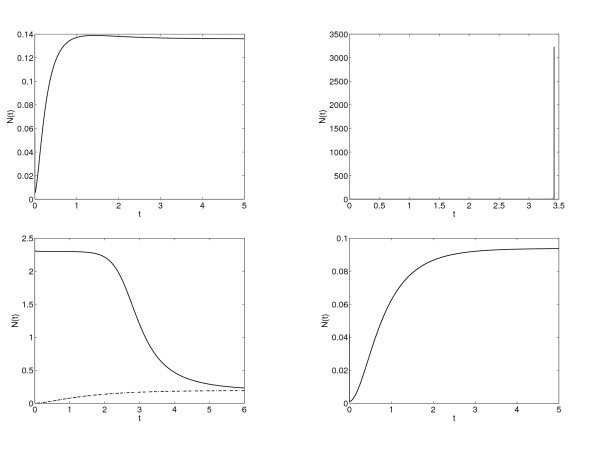
Firing rates N(t) for a≡1. Top left: b=0.5 with initial data a Maxwellian with: $v_{0}=0$ and $\sigma_{o}^{2}=0.25$. Top right: b=3 with initial data a Maxwellian with: $v_{0}=-1$ and $\sigma_{o}^{2}=0.5$. Bottom left: b=1.5 considering two different initial data: a Maxwellian with: $v_{0}=-1$ and $\sigma_{o}^{2}=0.5$ and a profile given by the expression (3.2) with N=2.31901. Bottom right: b=−1.5 with initial data a Maxwellian with: $v_{0}=-1$ and $\sigma_{o}^{2}=0.5$. The top right case seems to depict a blow-up phenomena demonstrated in Theorem 2.2.

In Figure 3 we show the time evolution of the distribution function p(v,t), in the case of a≡1 and b=0.5 for which there is only one steady state according to Theorem 3.1, considering as initial data a Maxwellian with $v_{0}=0$ and $\sigma_{0}^{2}=0.25$ in (5.1). We observe that the solution after 3.5 time units numerically achieves the steady state with the imposed tolerance. The top left subplot in Figure 4 describes the time evolution of the firing rate, which becomes constant after some time. This clearly corresponds to the case of a unique locally asymptotically stable stationary state. Let us remark that in the right subplot of Figure 3, we can observe the Lipschitz behavior of the function at $V_{R}$ as it should be from the jump in the flux and thus on the derivative of the solutions and the stationary states, see Section 3. 

For b=1.5, we proved in Section 3 that there are two steady states. With our simulations we can conjecture that the steady state with larger firing rate is unstable. However the stationary solution with low firing rate is locally asymptotically stable. We illustrate this situation in the bottom left subplot in Figure 4. Starting with a firing rate close to the high stationary firing value, the solution tends to the low firing stationary value.

**Fig. 5:**
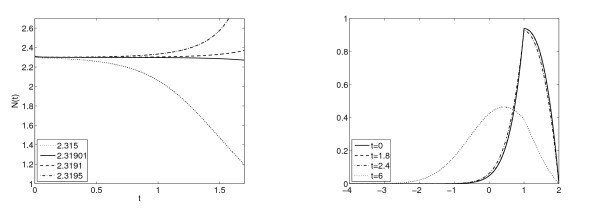
For b=1.5 and a(N)=1 figures show unstability of the steady state with higher firing rate. Left: Evolution on time of the firing rate considering different initial firing rate. Right: Evolution on time of the distribution function with initial firing rate 2.31901. In both figures we have considered $V_{R}=1$; $V_{F}=2$.

In Figure 5 we analyze in more details the behavior of the steady state with larger firing rate. The left subplot presents the evolution on time of the firing rate for different distribution function starting with profiles given by the expression (3.2) with *N* an approximate value of the stationary firing rate. We show that, depending of the initial firing rate considered, its behavior is different: tends to the lower steady state or goes to infinity. The firing rate for the solution with initial $N_{0}=2.31901$ remains almost constant for a period of time. Observe in Figure 5 that the difference between the initial data and the distribution function at time t=1.8 is almost negligible. However, the system evolves slowly and at t=6 the distribution is very close to the the lower steady state, see the bottom left subplot in Figure 4. 

In the bottom right subplot of Figure 4 we observe the evolution for a negative value of *b*, where we know that there is always a unique steady state, and its local asymptotic stability seems clear from the numerical experiments.

 The number of steady states is related with well-known neuronal phenomena, for instance, asynchronous behavior, when there exists only one stationary solution. Let us mention that bi- and multi-stable networks have been used to describe binocular rivalry in visual perception [24] and the process of decision making [25]. Our simulations show that the simple NNLIF model (1.4) describes networks with only one steady states and several stationary states, in terms of the connectivity parameter *b*.

**Fig. 6:**
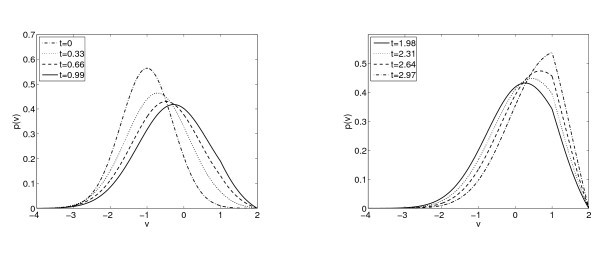
Distribution functions p(v,t) for a≡1 and b=3 at different times. See Figure 4 for the corresponding plots of N(t).

**Fig. 7:**
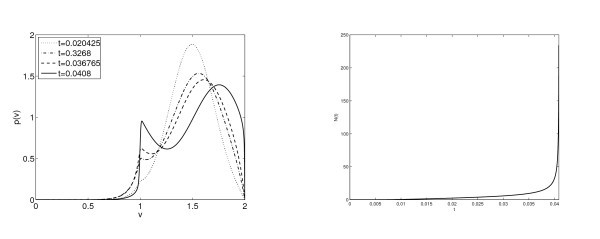
Parameter values are a≡1 and b=1.5 and this corresponds to two steady states. Left: Evolution of the distribution function p(v,t) in time of an initial Maxwellian centered at $v_{0}=1.5$ and with variance 0.005. Right: Time evolution of the firing rate; we observe numerically a blow-up behaviour for an initial data satisfying the condition of concentration around $V_{F}$ described in Theorem 2.2.

**Fig. 8:**
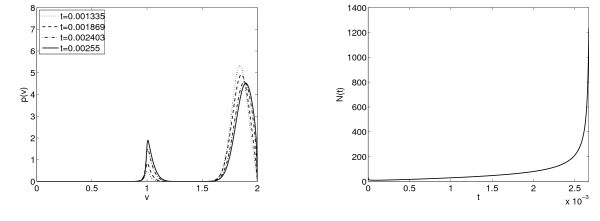
Parameter values are a≡1 and b=0.5 and this corresponds to a single steady state. Left: Evolution of the distribution function p(v,t) in time of an initial Maxwellian centered at $v_{0}=1.83$ and with variance 0.003. Right: Time evolution of the firing rate; again this seems to be a typical blow-up behaviour.

*No steady states. -* The results in Section 3 indicate that there are no steady states for b=3. In Figure 6 we observe the evolution on time of the distribution function *p* for this choice of the connectivity parameter *b*. In Figure 4 (right top) we show the time evolution of the firing rate, which seems to blow up in finite time. We observe how the distribution function becomes more and more picked at $V_{R}$ and $V_{F}$ producing an increasing value of the firing rate. The synchronization is a phenomenon that is of interest to neuroscientists, in these figures we do not observe it, but they do not show desynchronization either, since there are not steady states and no convergence of the firing rate. Therefore it could be possible to find periodic solutions which describe this phenomenon allowing for the formation of point Diracs in the firing rate. In this way, it could be related with the situation that we observe in Figures 7 and 8, where blow-up is analyzed, since the firing rate looks like tends to be a Dirac Delta. 

*Blow up. -* According to our blow-up Theorem 2.2, the blow-up in finite time of the solution happens for any value of b>0 if the initial data is concentrated enough on the firing rate. In Figures 7 and 8, we show the evolution on time of the firing rate with an initial data with mass concentrated close to $V_{F}$ for values of *b* in which there are either a unique or two stationary states. The firing rate increases without bound up to the computing time. It seems that the blow-up condition in Theorem 2.2 is not as restrictive as to say that the initial data is close to a Dirac Delta at $V_{F}$, as it is showed in Figure 7, where the initial condition is far from Dirac Delta at $V_{F}$. Let us finally mention that blow-up appears numerically also in case of $a(N)=a_{0}+a_{1}N$, but here the blow-up scenario is characterized by a break-up of the condition under which (1.6) has a unique solution *N*, that is,

$$a_{1}|\frac{\partial p}{\partial v}(V_{F},t)|<1.$$

Therefore, the blow-up in the value of the firing rate appears even if the derivative of *p* at the firing voltage does not diverge. This kind of behavior could be interpreted as a synchronization of a part of the network, since the firing rate tends to be a Dirac Delta.

## 6 Conclusion

The nonlinear noisy leaky integrate and fire (NNLIF) model is a standard Fokker-Planck equation describing spiking events in neuron networks. It was observed numerically in various places, but never stated as such, that a blow-up phenomena can occur in finite time. We have described a class of situations where we can prove that this happens. Remarkably, the system can blow-up for all connectivity parameter b>0, whatever is the (stabilizing) noise.

The nature of this blow-up is not mathematically proved. Nevertheless, our estimates in Lemma 2.3 indicate that it should not come from a vanishing behaviour for v≈−∞, or a lack of fast decay rate because the second moment in *v* is controlled uniformly in blow-up situations. Additionally, numerical evidence is that the firing rate N(t) blows-up in finite time whenever a singularity in the system occurs. This scenario is compatible with all our theoretical knowledge on the NNLIF and in particular with $L^{1}$ estimates on the total network activity (firing rate N(t)). Further understanding of the nature of blow-up behavior, and possible continuation, is a challenging mathematical issue. This blow-up phenomenon could be related to synchronization of the network therefore an interpretation in terms of neurophysiology would be interesting.

On the other hand, we have established that the set of steady states can be empty, a single state or two states depending on the network connectivity. These are all compatible with blow-up profile, and when they exist, numerics can exhibit convergence to a steady state, that is, to an asynchronous state for the network. Besides better understanding of the blow-up phenomena, several questions are left open; is it possible to have triple or more steady states? Which of them are stable? Can a bifurcation analysis help to understand and compute the set of steady states?

## Appendix

 This appendix is devoted to prove a Hardy-Poincaré’s-like inequality more general that the one stated in Proposition 4.3. Given m,n>0 on (0,∞) such that $\int_{0}^{\infty }m(y)\;dy=1$, we want to show that for all functions *f* on (0,∞), such that $\int_{0}^{\infty }m(y)f(y)\;dy=0$, the following inequality holds:

(7.1) $$\int_{0}^{\infty }m(x){\left|f(x)\right|}^{2}\;dx\leq A\int_{0}^{\infty }n(x){\left|f^{\prime }(x)\right|}^{2}\;dx,$$

provided all integrals make sense. Proposition 4.3 follows by considering $m(x)=n(x)=K\min(x,e^{-x^{2}/2})$, where *K* is a constant in such a way that $\int_{0}^{\infty }m(y)\;dy=1$ and parameterizing the interval from $\left(-\infty ,V_{F}\right]$ instead of [0,∞). Note that $p_{\infty }$ is equivalent to the function *m* both at 0 and at ∞ in terms of asymptotic behavior. We point out that for this kind of functions, $m(x)=n(x)=K\min(x,e^{-x^{2}/2})$, the Muckenhoupt’s criterion for Poincare’s inequality or their variants in [32,33] cannot be used, since 1/n(x) is not integrable at zero. However, the inequality (7.1) holds provided that $\int_{0}^{\infty }m(y)f(y)\;dy=0$. The main ingredient in the proof of (7.1) is to write

$$\frac{1}{2}f{\left(x\right)}^{2}=\frac{1}{2}{\left(\int_{0}^{\infty }m(y)(f(x)-f(y))\;dy\right)}^{2},$$

where we used $\int_{0}^{\infty }m(y)f(y)\;dy=0$ and $\int_{0}^{\infty }m(y)\;dy=1$. In this way we can consider two functions, to be chosen later, ϕ,ψ>0 to obtain

$$\begin{array}{rl} \frac{1}{2}f{\left(x\right)}^{2}= & \frac{1}{2}{\left(\int_{0}^{\infty }m(y)(f(x)-f(y))\;dy\right)}^{2}\\ \leq & {\left(\int_{0}^{x}m(y)\int_{y}^{x}f^{\prime }(z)\;dz\;dy\right)}^{2}+{\left(\int_{x}^{\infty }m(y)\int_{x}^{y}f^{\prime }(z)\;dz\;dy\right)}^{2}\\ = & {\left(\int_{0}^{x}f^{\prime }(z)\int_{0}^{z}m(y)\;dy\;dz\right)}^{2}+{\left(\int_{x}^{\infty }f^{\prime }(z)\int_{z}^{\infty }m(y)\;dy\;dz\right)}^{2}\\ \leq & \int_{0}^{x}f^{\prime }{\left(z\right)}^{2}n(z)\phi (z)\;dz\;\int_{0}^{x}\frac{{\left(\int_{0}^{z}m(y)\;dy\right)}^{2}}{n(z)\phi (z)}\;dz\\ & +\int_{x}^{\infty }f^{\prime }{\left(z\right)}^{2}n(z)\psi (z)\;dz\;\int_{x}^{\infty }\frac{{\left(\int_{z}^{\infty }m(y)\;dy\right)}^{2}}{n(z)\psi (z)}\;dz. \end{array}$$

Therefore, we have

$$\frac{1}{2}\int_{0}^{\infty }m(x){\left|f(x)\right|}^{2}\;dx\leq I+\mathrm{II},$$

where, using the notation, $M(x)=\int_{0}^{x}m(y)\;dy$

$$I=\int_{0}^{\infty }m(x)\int_{0}^{x}f^{\prime }{\left(z\right)}^{2}n(z)\phi (z)\;dz\;\int_{0}^{x}\frac{M{\left(y\right)}^{2}}{n(y)\phi (y)}\;dy\;dx$$

and

$$\mathrm{II}=\int_{0}^{\infty }m(x)\int_{x}^{\infty }f^{\prime }{\left(z\right)}^{2}n(z)\psi (z)\;dz\;\int_{x}^{\infty }\frac{{\left(1-M(y)\right)}^{2}}{n(y)\psi (y)}\;dy\;dx.$$

To conclude the proof we analyse both terms. Considering Fubini’s Theorem we obtain

$$\begin{array}{rl} I= & \int_{0}^{\infty }m(x)\int_{0}^{x}f^{\prime }{\left(z\right)}^{2}n(z)\phi (z)\;dz\;\int_{0}^{x}\frac{M{\left(y\right)}^{2}}{n(y)\phi (y)}\;dy\;dx\\ = & \int_{0}^{\infty }f^{\prime }{\left(z\right)}^{2}n(z)\phi (z)\int_{z}^{\infty }m(x)\int_{0}^{x}\frac{M{\left(y\right)}^{2}}{n(y)\phi (y)}\;dy\;dx\;dz\\ \leq & A_{1}\int_{0}^{\infty }f^{\prime }{\left(z\right)}^{2}n(z)\;dz \end{array}$$

with

(7.2) $$A_{1}:=\underset{z>0}{\sup}\phi (z)\int_{0}^{\infty }\frac{M{\left(y\right)}^{2}}{n(y)\phi (y)}(1-M(y\vee z))\;dy\;\text{where }y\vee z\equiv \max(y,z),$$

and

$$\begin{array}{rl} \mathrm{II}= & \int_{0}^{\infty }m(x)\int_{x}^{\infty }f^{\prime }{\left(z\right)}^{2}n(z)\psi (z)\;dz\;\int_{x}^{\infty }\frac{{\left(1-M(y)\right)}^{2}}{n(y)\psi (y)}\;dy\;dx\\ = & \int_{0}^{\infty }f^{\prime }{\left(z\right)}^{2}n(z)\psi (z)\int_{0}^{z}m(x)\int_{x}^{\infty }\frac{{\left(1-M(y)\right)}^{2}}{n(y)\psi (y)}\;dy\;dx\;dz\\ \leq & A_{2}\int_{0}^{\infty }f^{\prime }{\left(z\right)}^{2}n(z)\;dz \end{array}$$

with

(7.3) $$A_{2}:=\underset{z>0}{\sup}\psi (z)\int_{z}^{\infty }\frac{{\left(1-M(y)\right)}^{2}}{n(y)\psi (y)}M(y\wedge z)\;dy\;\text{where}\;y\wedge z\equiv \min(y,z).$$

To conclude the proof, it remains to choose the functions *ϕ* and *ψ* such that, $A_{1},A_{2}<\infty$. Using L’Hôpital’s rule and, after some tedious but easy computations and calculus arguments, we obtain that in the case at hand, $m(x)=n(x)=K\min(x,e^{-x^{2}})$, we can take $\phi (x)=\psi (x)=1+\sqrt{x}$.

## Competing interests

The authors declare that they have no competing interests.
